# MODIFIED MUBARAK TECHNIQUE FOR FLEXIBLE FLATFOOT CORRECTION IN CHILDREN AND ADOLESCENTS

**DOI:** 10.1590/1413-785220233104e265045

**Published:** 2023-07-31

**Authors:** BRUNO AIR MACHADO DA SILVA, NILZIO ANTÔNIO DA SILVA, JONATAS BARBOSA VASCONCELOS

**Affiliations:** 1 Hospital de Urgencias de Aparecida de Goiania, Goiania, GO, Brazil.; 2 Instituto Ortopedico de Goiania, Goiania, GO, Brazil.; 3 Universidade Federal de Goiania, Escola de Medicina, Departamento de Reumatologia, Goiania, GO, Brazil.

**Keywords:** Flatfoot, Tarsal Bones, Osteotomy, Pé Chato, Ossos do Tarso, Osteotomia

## Abstract

**Objective:**

To describe the technique, analyze possible radiographic correction and evaluate the clinical result of medial and plantar calcaneal displacement osteotomy associated with opening wedge cuboid osteotomy for flexible flatfoot correction.

**Methods:**

23 patients (30 feet) diagnosed with flexible flat foot treated with plantar and medial calcaneal displacement osteotomy associated with opening wedge cuboid osteotomy were evaluated retrospectively. In the lateral radiographs calcaneal pitch and Meary’s angle were the radiographic parameters evaluated; while the talonavicular coverage angle was evaluated in the anteroposterior radiographs. To assess the clinical outcome of the surgical procedure, the American Orthopedic Foot and Ankle Society Score (AOFAS) for the ankle and hindfoot was adopted.

**Results:**

The mean values of the evaluated angles and AOFAS score for ankle and hindfoot significantly improved when comparing pre- and postoperative values.

**Conclusion:**

Plantar and medial calcaneal displacement osteotomy associated with an opening wedge cuboid osteotomy is able to improve radiological and clinical parameters of child patients with flexible flatfoot. ***Level of Evidence III, Retrospective Comparative Study.***

## RESUMO

**Objetivo:**

Descrever a técnica, analisar possíveis correções radiográficas e avaliar o resultado clínico da osteotomia de deslocamento medial e plantar do calcâneo associada à osteotomia em cunha de adição do cuboide para correção do pé plano flexível de crianças.

**Métodos:**

Foram avaliados retrospectivamente 23 pacientes (30 pés) com diagnóstico de pé plano flexível tratadas com osteotomia de deslocamento plantar e medial do calcâneo associada à osteotomia em cunha de adição do cuboide. Os parâmetros radiográficos avaliados nas imagens em perfil foram o pitch do calcâneo e o ângulo de Meary, enquanto nas radiografias anteroposteriores o ângulo de cobertura do tálus. Para avaliar o resultado clínico do procedimento cirúrgico, foi adotado o escore da American Orthopaedic Foot and Ankle Society (AOFAS) para tornozelo e retropé.

**Resultados:**

Os valores médios dos ângulos avaliados e do escore AOFAS para tornozelo e retropé melhoraram significativamente na comparação dos resultados pré e pós-operatórios.

**Conclusão:**

A osteotomia de deslocamento plantar e medial do calcâneo associada à osteotomia em cunha de adição do cuboide é capaz de melhorar os parâmetros radiológicos e clínicos de crianças com pé plano flexível. **Nível de Evidência III, Estudo Comparativo Restrospectivo.**

## INTRODUCTION

Flexible flatfoot is defined by the loss of the longitudinal arch of the foot and hindfoot valgus, in addition to abductus and a certain degree of supination of the forefoot relative to the midfoot. The deformity is assessed with the tip toe test or the Jack test.^1^

If the patient does not respond to conservative treatment, surgery is indicated. Recently, many surgical procedures have been described, among which osteotomies have been the treatment of choice for children with flexible flat feet since it does not sacrifice foot mobility.^2^

In 1893, Gleich described medialization calcaneal osteotomy to correct hindfoot valgus,^3^ which was later popularized by Koutsogiannis in the treatment of flexible flatfoot.^4^ However, it cannot restore the longitudinal arch of the foot.^4^

The concept of correcting valgus flat foot with lateral column lengthening osteotomy was achieved by Evans. In 1975, Evans described that, when the lateral wall of the calcaneus was elongated, the navicular moved medially, improving both talar coverage and the longitudinal arch of the foot.^5^

A few years later, Mubarak described calcaneal-cuboid-cuneiform osteotomy to correct a child’s planovalgus foot. The calcaneal osteotomy corrects hindfoot valgus, the opening wedge in the cuboid lengthens the lateral column realigning the talonavicular joint, while the cuneiform osteotomy allows forefoot plantar flexion.^6^ ^)^ It was with Mubarak’s concept that we associated the opening wedge cuboid osteotomy and modified the calcaneal osteotomy by displacing it both medially and plantarly.

Based on Mubarak’s concept, a plantar and medial calcaneal displacement osteotomy associated with an opening wedge cuboid was proposed for treating flexible flat foot in children.

Our hypothesis is that the plantar and medial calcaneal displacement is sufficient to correct the plantar arch and hindfoot valgus and that the opening wedge cuboid can correct forefoot abductus. Cuneiform osteotomy would not be necessary.

This study aims to describe the technique, analyze possible radiographic correction and evaluate the clinical result.

## METHODS

The research project was evaluated by the Research Ethics Committee of the institution and approved under opinion number 2,160,581 and registered on Plataforma Brasil, protocol CAAE number: 68282217.2.0000.5078.

We retrospectively evaluated 23 patients (30 feet) diagnosed with flexible flat foot treated with plantar and medial calcaneal displacement osteotomy associated with opening wedge cuboid osteotomy. The surgery was performed by a single surgeon from 2013 to 2016, with at least two years of follow-up.

Inclusion criteria were patients with symptomatic flexible flat foot, aged 10-18 years with follow-up of at least two years. Patients with tarsal coalition, rigid flat foot, posterior tibial dysfunction, and patients undergoing other foot bone surgeries were excluded.

The flexible flat foot diagnosis was based on loss of the longitudinal plantar arch and hindfoot valgus with the patient in orthostatism. Flexibility was defined as the reconstitution of the longitudinal plantar arch of the foot and calcaneus varus with the patient on tiptoe.

Initially, conservative treatment was attempted by changing shoes, using insole, and muscle stretching of the posterior chain muscles of the leg. Surgical treatment was indicated to patients who remained in pain after 6 months of conservative measures.

Patients underwent radiographic examination of the feet in the preoperative period, 6 weeks after surgery, and at the last office visit. The parameters evaluated in the lateral radiographs were the calcaneal pitch and the Meary’s angle; whereas in the anteroposterior radiographs, the talonavicular coverage angle was evaluated, following Davids, Gibson, and Pugh.^7^ The measurements were taken by the senior author (B.A.M), using the WTT-Dicom Viewer version 0.5.326 program.

To assess the clinical outcome of the surgical procedure, the American Orthopedic Foot and Ankle Society Score (AOFAS) for the ankle and hindfoot was adopted. The scores measured were considered excellent if ranging 90-100, good if ranging 80-89, fair if ranging 70-79, and poor if it had less than 70 points.^8^

Data were tabulated in a spreadsheet using Excel program (Office 2013) and later analyzed using the statistical package Statistical Package of Social Sciences (SPSS 24.0). Data normality was verified using the Shapiro-Wilk test. The comparison of AP - COB TALUS, P - PITCH and P - MEARY values before and after treatment was performed using the paired t-test. In all analyses, a 5% significance level (p < 0.05) was adopted.

### Surgical technique

The procedures were performed with the patient under spinal anesthesia and a 300 mmHg tourniquet at the thigh level.

**Figure 1:**
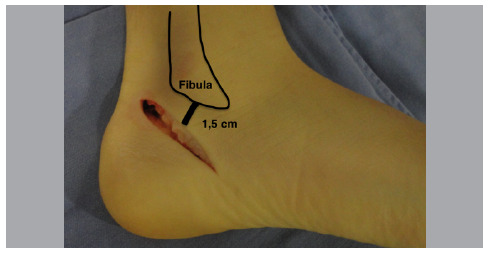
Calcaneal osteotomy approach.

A 1.5 cm access from the distal tip of the fibula is made obliquely (45° with the ground) starting from the upper edge of the calcaneus to the lower edge of the distal part of the calcaneus (Figure 1).

The sural nerve is moved dorsally. A calcaneal osteotomy is then performed, respecting the 45° angle with the ground. Initially, an oscillatory saw is used, and the medial wall of the calcaneus is cut with an osteotome.

**Figure 2:**
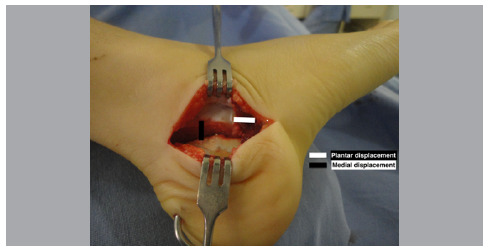
Calcaneal osteotomy.

The posterior fragment of the calcaneus is medialized until its medial border is aligned with the talar sustentaculum, a displacement of approximately 5-10 mm. Additionally, a plantar deviation of this same fragment is made around 5-10 mm. The calcaneal osteotomy is then fixed with 02 k-wire (Figure 2).

**Figure 3:**
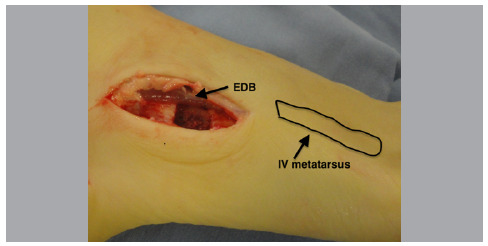
Cuboid approach.

Once the calcaneus is fixed, a lateral access is made over the cuboid, plantarly to the extensor digitorum brevis (EDB) in alignment with the IV metatarsal (Figure 3).

**Figure 4:**
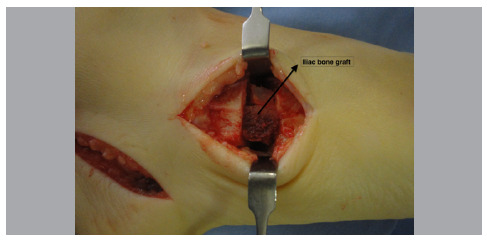
Opening wedge cuboid osteotomy.

The EDB is moved dorsally and then an opening wedge cuboid osteotomy is performed equidistant from the calcaneal-cuboid and cuboid-metatarsal joints. A spreader is placed on the osteotomy to make room for placement of a structured bone graft taken from the iliac crest, approximately 10 mm thick (Figure 4).

**Figure 5:**
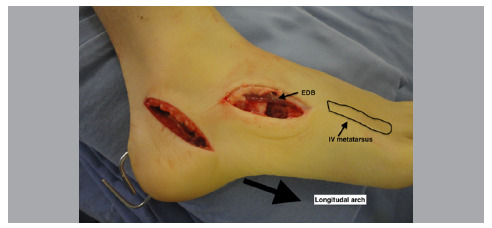
Final aspect of foot.

No fixation is used for this osteotomy. Once the procedure is over, it is possible to see the formation of the longitudinal plantar arch (Figure 5)

### Post operative management

**Figure 6:**
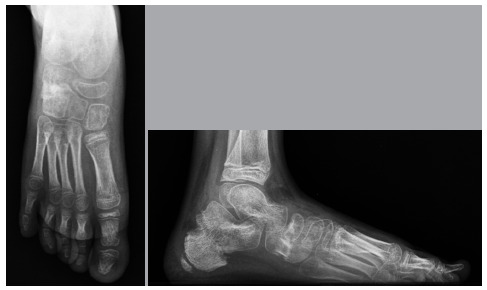
A: Anteroposterior post-op radiograph; B: Lateral radiograph.

The patient usually stays at the hospital the day after surgery. Knee walking boot is recommended to protect the osteotomies, and patients were kept non-weight bearing for 6 weeks. A radiological evaluation is performed after six weeks and, according to the result, K pins are removed. Weight-bearing is then allowed with boot on for 2 weeks. The patient is released to wear shoes eight weeks after surgery and once physical therapy rehabilitation is started. A new radiographic evaluation is performed after 12 weeks of surgery (Figure 6A and 6B).

## RESULTS

Of the 27 selected patients (36 feet) operated during the chosen period, 4 cases were excluded from the study: 2 patients with tarsal coalition, 1 patient with cerebral palsy (2 feet), 1 case of review of failure of previous surgical treatment, and 1 case without radiography. The total number of patients included in this study was 23 (30 feet).

We evaluated the 23 (13 boys and 10 girls) patients, diagnosed with flexible flat foot in childhood, using medial and plantar calcaneal displacement osteotomy associated with opening wedge cuboid osteotomy at our referral center from 2013 to 2016. The mean age of girls at the time of surgery was 12.7 ± 2.3 years, and the boys were 10.5 ± 1.8 years. The mean follow-up time of patients after surgery was 37 ± 4.8 months (Table 1).

**Table 1:** Baseline data of all patients (N = 23).

Characteristic	N (%)
Total patients	23
Boys	13 (56.5)
Girls	10 (43.5)
Mean age	
Boys	10.5
Girls	12.7

All osteotomies consolidated in eight weeks. No loss of correction was observed in any patient during follow-up (26-60 months). The mean values of the evaluated angles and AOFAS score for ankle and hindfoot significantly improve when comparing pre and postoperative values (Table 2).

**Table 2:** Preoperative and postoperative measures.

	(Mean ± Standard deviation)		p*
	Pre	Post	
Talonavicular coverage angle	38.83 ± 13.40	22.49 ± 13.35	< 0.001
Calcaneal pitch	12.25 ± 4.07	23.75 ± 4.05	< 0.001
Meary's angle	18.90 ± 7.76	8.41 ± 6.16	< 0.001
AOFAS score	62 ± 11.1	89.8 ± 3.7	< 0.001

* Student’s t-test.

The postoperative complications observed were superficial infection (one patient), suture dehiscence (one patient) and k-wire path infection (two patients). No subluxation of the calcaneal-cuboid joint or lateral foot pain was observed.

## DISCUSSION

Surgical treatment for flexible flat foot in children is indicated after a failed attempt with conservative treatment. Surgery aims to relief pain in the medial plantar surface of the midfoot and/or in the sinus tarsus, which interferes with the patient’s day-to-day activities. Among surgical interventions, osteotomies have become the first choice due to the possibility of realigning the foot without sacrificing its movements.^2^

Rathjen and Mubarak described sliding and medial closing wedge osteotomy of the calcaneus associated with osteotomy of plantar closing wedge of the cuneiform and lateral opening wedge in the cuboid (triple “C”). This technique proved to be capable of correcting the deformities found in the flexible flat foot of children, with good functional results and a low complication rate.^6^

When comparing the technique proposed by Mubarak and the isolated lengthening of the external column of the foot (Evans modified by Mosca), it was observed that the osteotomy of lengthening the lateral column has greater power to correct the talar coverage and the talus-first metatarsal angle on anteroposterior radiograph of the foot. Confirming a better correction of the foot abductus with the Evans technique modified by Mosca.^2^

However, lengthening the lateral column presents a greater chance of subluxation of the calcaneal-cuboid joint (possibly increasing chances of arthrosis), a higher complication rate (18.2% × 10%), pain at the lateral edge of the foot, a chance of migration of the bone graft and possible injury to the calcaneal joint surface.^8^

The association of plantar closing wedge osteotomy of the medial cuneiform with the aforementioned intervention aims to restore the longitudinal arch of the foot.^6^ Although this association has proven capable of restoring radiological parameters, it increases surgical time, implies additional surgical incision and leads to a shortening of the medial column of the foot.

Our work showed that it is possible to correct the deformities found in the flexible flat foot of children, including the restoration of the longitudinal plantar arch of the foot without plantar closing wedge osteotomy of the medial cuneiform. Plantar and medial calcaneal displacement osteotomy associated with opening wedge cuboid osteotomy was able to improve talonavicular coverage, calcaneal pitch, and Meary’s angle.

When we compared the triple “C” technique with medial and plantar calcaneal displacement associated with opening wedge cuboid osteotomy, we noticed similar radiological results. In the work by Mubarak et al.,^2^ the talus-first metatarsal angle changed from 21.8 ± 9.3 to 15.5 ± 11.1, talonavicular coverage angle changed from 41 ± 9.2 to 28 ± 14.7, and Meary’s angle changed from 25.3 ± 12.2 to 16.1 ± 10.25. All three parameters mentioned showed a statistically significant improvement (p < 0.05). In the surgical technique described by our group, we found that the same aforementioned parameters showed a statistically significant improvement.

**Figure 7:**
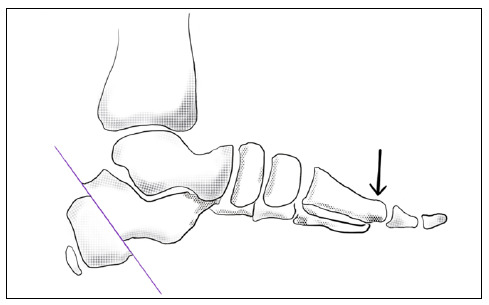
Effect calcaneus osteotomy on Meary’s angle.

Like Mubarak, we performed the lengthening of the external column of the foot through the opening wedge cuboid osteotomy, however we were able to improve Meary’s angle without the medial cuneiform osteotomy. The improvement in this angle can be explained by the tendency of the 1st ray to flex plantarly as a result of the medial and plantar calcaneal displacement osteotomy (Figure 7).

The medial and plantar calcaneal displacement osteotomy associated with cuboid opening wedge proved to be able to improve patients’ clinical condition. Patients showed an improvement in the AOFAS scale from poor to good, corroborating the outcomes of other studies related to “triple C” osteotomy.^8^ ^)-(^ ^10^

Our work has some limitations: the small number of patients, a short follow-up time without a control group, and the retrospective study model. A prospective and randomized study with a control group and longer follow-up is necessary. Moreover, it is necessary to prove the effect of plantar and medial calcaneal displacement on the first ray, possibly with weight-bearing tomography.

## CONCLUSION

Our work showed that the plantar and medial calcaneal displacement osteotomy associated with opening wedge cuboid osteotomy can improve radiological and clinical parameters of flexible flat feet in children.
